# Association between vision impairment and mortality: a systematic review and meta-analysis

**DOI:** 10.1016/S2214-109X(20)30549-0

**Published:** 2021-02-16

**Authors:** Joshua R Ehrlich, Jacqueline Ramke, David Macleod, Helen Burn, Chan Ning Lee, Justine H Zhang, William Waldock, Bonnielin K Swenor, Iris Gordon, Nathan Congdon, Matthew Burton, Jennifer R Evans

**Affiliations:** a Department of Ophthalmology and Visual Sciences, and Institute for Healthcare Policy and Innovation, University of Michigan, Ann Arbor, MI, USA; b MRC Tropical Epidemiology Group, London School of Hygiene & Tropical Medicine, London, UK; c International Centre for Eye Health, London School of Hygiene & Tropical Medicine, London, UK; d School of Optometry and Vision Science, University of Auckland, Auckland, New Zealand; e Department of Ophthalmology, Stoke Mandeville Hospital, Aylesbury, UK; f St Paul's Eye Unit, Royal Liverpool University Hospital, Liverpool, UK; g Manchester Royal Eye Hospital, Manchester, UK; h University of Cambridge School of Medicine, Cambridge, UK; i Wilmer Eye Institute, Johns Hopkins University School of Medicine, Baltimore, MD, USA; j Department of Epidemiology, Johns Hopkins Bloomberg School of Public Health, Baltimore, MD, USA; k Queen's University Belfast, Belfast, UK; l Zhongshan Ophthalmic Center, Guangzhou, China; m Moorfields Eye Hospital, London, UK

## Abstract

**Background:**

The number of individuals with vision impairment worldwide is increasing because of an ageing population. We aimed to systematically identify studies describing the association between vision impairment and mortality, and to assess the association between vision impairment and all-cause mortality.

**Methods:**

For this systematic review and meta-analysis, we searched MEDLINE (Ovid), Embase, and Global Health database on Feb 1, 2020, for studies published in English between database inception and Feb 1, 2020. We included prospective and retrospective cohort studies that measured the association between vision impairment and all-cause mortality in people aged 40 years or older who were followed up for 1 year or more. In a protocol amendment, we also included randomised controlled trials that met the same criteria as for cohort studies, in which the association between visual impairment and mortality was independent of the study intervention. Studies that did not report age-adjusted mortality data, or that focused only on populations with specific health conditions were excluded. Two reviewers independently assessed study eligibility, extracted the data, and assessed risk of bias. We graded the overall certainty of the evidence using the Grading of Recommendations, Assessment, Development and Evaluations framework. We did a random-effects meta-analysis to calculate pooled maximally adjusted hazard ratios (HRs) for all-cause mortality for individuals with a visual acuity of <6/12 versus those with ≥6/12; <6/18 versus those with ≥6/18; <6/60 versus those with ≥6/18; and <6/60 versus those with ≥6/60.

**Findings:**

Our searches identified 3845 articles, of which 28 studies, representing 30 cohorts (446 088 participants) from 12 countries, were included in the systematic review. The meta-analysis included 17 studies, representing 18 cohorts (47 998 participants). There was variability in the methods used to assess and report vision impairment. Pooled HRs for all-cause mortality were 1·29 (95% CI 1·20–1·39) for visual acuity <6/12 versus ≥6/12, with low heterogeneity between studies (n=15; τ^2^=0·01, *I*^2^=31·46%); 1·43 (1·22–1·68) for visual acuity <6/18 versus ≥6/18, with low heterogeneity between studies (n=2; τ^2^=0·0, *I*^2^=0·0%); 1·89 (1·45–2·47) for visual acuity <6/60 versus ≥6/18 (n=1); and 1·02 (0·79–1·32) for visual acuity <6/60 versus ≥6/60 (n=2; τ^2^=0·02, *I*^2^=25·04%). Three studies received an assessment of low risk of bias across all six domains, and six studies had a high risk of bias in one or more domains. Effect sizes were greater for studies that used best-corrected visual acuity compared with those that used presenting visual acuity as the vision assessment method (p=0·0055), but the effect sizes did not vary in terms of risk of bias, study design, or participant-level factors (ie, age). We judged the evidence to be of moderate certainty.

**Interpretation:**

The hazard for all-cause mortality was higher in people with vision impairment compared with those that had normal vision or mild vision impairment, and the magnitude of this effect increased with more severe vision impairment. These findings have implications for promoting healthy longevity and achieving the Sustainable Development Goals.

**Funding:**

Wellcome Trust, Commonwealth Scholarship Commission, National Institutes of Health, Research to Prevent Blindness, the Queen Elizabeth Diamond Jubilee Trust, Moorfields Eye Charity, National Institute for Health Research, Moorfields Biomedical Research Centre, Sightsavers, the Fred Hollows Foundation, the Seva Foundation, the British Council for the Prevention of Blindness, and Christian Blind Mission.

## Introduction

Over half a billion people are blind or have distance vision impairment worldwide.^1^ Blindness and vision impairment are most common among adults aged 50 years and older, who account for more than 80% of people with vision loss.^2^ As populations continue to age, the prevalence of vision impairment and blindness are projected to more than double over the next 30 years.^1^ The impacts of vision impairment and blindness are wide-reaching, including an increased risk of falls, cognitive impairment and dementia, depression, disability, and loss of independence.^2^, ^3^ Some studies have also reported that vision impairment and blindness are associated with an increased risk of mortality.^4^

**Research in context**

**Evidence before this study**

We searched PubMed on Feb 1, 2020, for primary research articles published in English from database inception up to Feb 1, 2020. A full list of the search terms used can be found in the appendix (pp 2–5). We identified a single meta-analysis of the association between vision impairment and mortality. This study analysed the association between vision impairment (measured by use of objective clinical instruments, self-reported visual difficulty, and administrative claims) and all-cause mortality. The results showed a significant association between mortality and the highest degree of vision impairment when compared with no vision impairment. The study did not include a narrative review of the literature, an assessment of the risk of bias in included studies, or an overall grading of the certainty of the evidence. We also found that, since the publication of this meta-analysis, several additional primary research articles had been published, and that some of these articles were from regions of the world, including sub-Saharan Africa and east Asia, that were previously not well represented in the literature.

**Added value of this study**

This systematic review and meta-analysis adds to the existing literature by including newly published articles investigating the association between vision impairment and all-cause mortality in adults worldwide. By conducting a full systematic review, we have identified opportunities for standardisation of data collection and reporting, and we found additional studies on the topic that could not be included in the meta-analysis due to the choice of vision impairment thresholds, the analytic methods used, or both. Additionally, we used the Quality in Prognostic Studies tool to assess the risk of bias in included studies, and the Grading of Recommendations, Assessment, Development and Evaluations (GRADE) framework to judge the overall certainty of the evidence. We also did meta-analyses comparing the hazard of all-cause mortality in participants with and without specified levels of visual acuity impairment. We found that the hazard of mortality was higher among participants with mild vision impairment (visual acuity <6/12) compared with those who had no vision impairment (≥6/12), and was higher in those with moderate vision impairment (<6/18) compared with those with no vision impairment or mild vision impairment (≥6/18). Among people with severe vision impairment or blindness (visual acuity <6/60), the hazard for mortality was higher than for those with normal vision or mild vision impairment. However, no association between vision impairment and mortality was observed when participants with a visual acuity of worse than 6/60 were compared with those with visual acuity of better than 6/60, probably because the reference group (ie, those with a visual acuity of ≥6/60) comprised a heterogeneous group of participants with moderate vision impairment, mild vision impairment, and normal vision. We assessed the robustness of our findings by examining heterogeneity in our effect estimates, performing meta-regressions, and testing for publication bias; we found little heterogeneity in our estimates and no evidence of publication bias. However, studies reported a significantly larger effect size if they assessed the association between mortality and best-corrected visual acuity rather than the association between mortality and presenting visual acuity.

**Implications of all the available evidence**

Our systematic review and meta-analysis highlights the prevailing finding that vision impairment is associated with a higher hazard of age-adjusted all-cause mortality in adults across diverse global settings and populations. Using the GRADE framework, we are moderately confident that the mortality risk associated with vision impairment reported in this study is likely to be close to the true value, but there is a possibility that the true hazard might be substantially different. Future research should focus on assessing the association between mortality and other clinical measures of vision (eg, visual field or contrast sensitivity) that have been shown to affect functioning, quality of life, and health outcomes. In addition, no studies on this topic have been conducted in eastern Europe, Latin America, the Caribbean, north Africa, or the Pacific islands, and data from these regions is important to improve the generalisability of study findings. Future calculations of disability-adjusted life-years might include years of life lost due to vision impairment, which could provide a more complete estimate of the overall global burden of vision impairment. As most vision impairment and blindness is avoidable or correctable, this study has important implications for optimising healthy longevity for populations worldwide, and for achieving the Sustainable Development Goals (SDGs), particularly SDG3, which aims to “ensure healthy lives and promote well-being for all at all ages”.

In a previous meta-analysis, Zhang and colleagues^4^ examined 29 studies that measured the association between vision impairment and mortality. Among these studies, 15 used objective measures of vision (eg, visual acuity), whereas others relied on self-reported visual difficulty, or vision impairment defined by International Classification of Diseases (ICD) codes. The risk of bias in these studies and the overall quality of evidence was not assessed.^4^ Since this meta-analysis was published in 2016, several additional primary studies have been published,^5^, ^6^, ^7^, ^8^, ^9^, ^10^ including those done in previously under-represented regions, such as sub-Saharan Africa^5^ and east Asia.^6^

An improved understanding of the association between vision impairment and mortality is needed to inform public policy, public health planning, and allocation of limited health-care resources. As part of *The Lancet Global Health* Commission on Global Eye Health,^11^ we therefore did an updated systematic review and meta-analysis to assess the extent, strength, and quality of evidence on the association between vision impairment and age-adjusted all-cause mortality in adults worldwide. To provide a comprehensive assessment of the current state of scientific knowledge, we also examined the potential causes of variation in this association.

## Methods

### Search strategy and selection criteria

In this systematic review and meta-analysis, we searched MEDLINE (Ovid), Embase, and Global Health database on Feb 1, 2020, for studies published in English between database inception and Feb 1, 2020. We included prospective and retrospective cohort studies that measured the association between vision impairment and all-cause mortality in people aged 40 years or older, who were followed up for 1 year or more. In a protocol amendment, we included randomised controlled trials (RCTs), as long as the reported association between vision impairment and mortality was independent of the study intervention; we also included RCTs and cohort studies with participants younger than 40 years if more than 50% of participants were aged 40 years or older. We assessed the effect of these protocol amendments on effect estimates in meta-regression analyses. Conference abstracts and grey literature were not included. We identified additional studies by searching the reference lists of included studies. The searches were done by an information specialist (IG), and the search strategy and full list of search terms used are provided in the appendix (pp 2–5).

We intended to include studies in which vision was assessed by use of any objective clinical measure of vision and in which age-adjusted all-cause mortality was reported. We only included studies in the meta-analysis that assessed visual acuity, as few studies reported associations with other measures of vision (eg, contrast sensitivity or visual fields). In studies that used best-corrected visual acuity and presenting visual acuity as vision assessment methods, data on best-corrected visual acuity were included in the primary analysis. Studies that did not report age-adjusted mortality, or that focused only on populations with specific health conditions (eg, diabetes or stroke) were excluded, as in such cases, age and systemic disease might have a strong confounding effect.

The internet-based systematic review management software, Covidence (Veritas Health Innovation, Melbourne, VIC, Australia), was used to screen titles and abstracts, assess full-text articles, and extract summary estimates from included studies. All titles, abstracts, and full-text articles were screened independently by pairs of investigators (one of JREh, JR, and JREv paired with one of HB, CNL, JHZ, or WW). Disagreements were resolved through discussion and adjudication by a third investigator (JREh, JR, or JREv), as needed.

This study was done as part of *The Lancet Global Health* Commission on Global Eye Health.^11^ The complete study protocol was registered prospectively at the Open Science Framework Registries, and has been published previously.^12^ Amendments to the initial study protocol are noted herein. We used the PROGRESS prognosis research strategy^13^ to develop the protocol for this study, which is reported according to the Preferred Reporting Items for Systematic Reviews and Meta-Analyses guidelines (appendix pp 6–7).^14^

### Data analysis

The following data were extracted from each publication: study setting; study timing; study design; sample size; age, gender, and ethnicity of study participants; follow-up time; definition of vision impairment; methods and eyes used for vision assessment; methods of mortality assessment; statistical modelling approach; and effect size estimates. Three pairs of investigators (JREh and CNL, JR and JHZ, and JREv and HB) independently extracted data from each article, guided by the Critical Appraisal and Data Extraction for Systematic Reviews of Prediction Modelling Studies framework.^15^ Disagreements were resolved through discussion and adjudication by a third investigator (JREh, JR, or JREv), as needed. When duplicate data were available from multiple published studies, preference was given to the study with the longest follow-up. Many studies reported results from models with different combinations of covariates. When estimates were reported with more than one level of adjustment, we extracted two estimates: (1) the age-adjusted estimate with the fewest additional covariates (minimally adjusted); and (2) the age-adjusted estimate with the greatest number of additional covariates (maximally adjusted). When multiple publications contained data from a single cohort, data were extracted from the publication with the longest follow-up. When data on multiple cohorts were presented in a single publication, each cohort was separately eligible for inclusion.

The risk of bias in included studies was assessed independently by three pairs of investigators (JREh and CNL, JR and JHZ, and JREv and HB) using the Quality in Prognostic Studies (QUIPS) tool.^16^ The following domains were assessed: study participation, attrition, prognostic factor measurement, outcome measurement, confounding, and statistical analysis and reporting. Risk of bias was defined as high if a study received a high rating in one or more domains; low if it received a low rating in all six domains; or moderate if it did not meet criteria for low or high risk or bias. Disagreements on risk of bias ratings were resolved through discussion and adjudication by a third investigator (JREh, JR, or JREv).

We classified vision impairment according to WHO reporting standards: mild vision impairment (visual acuity <6/12 to 6/18); moderate vision impairment (<6/18 to 6/60); and severe vision impairment or blindness (<6/60). We compared the following visual acuity thresholds in the meta-analysis: (1) <6/12 versus ≥6/12; (2) <6/18 versus ≥6/18; (3) <6/60 versus ≥6/18; and (4) <6/60 versus ≥6/60. Note that some studies classified visual acuities at the category threshold as vision impairment, whereas others did not (eg, ≤6/12 *vs* 6/12).

**Figure 1:**
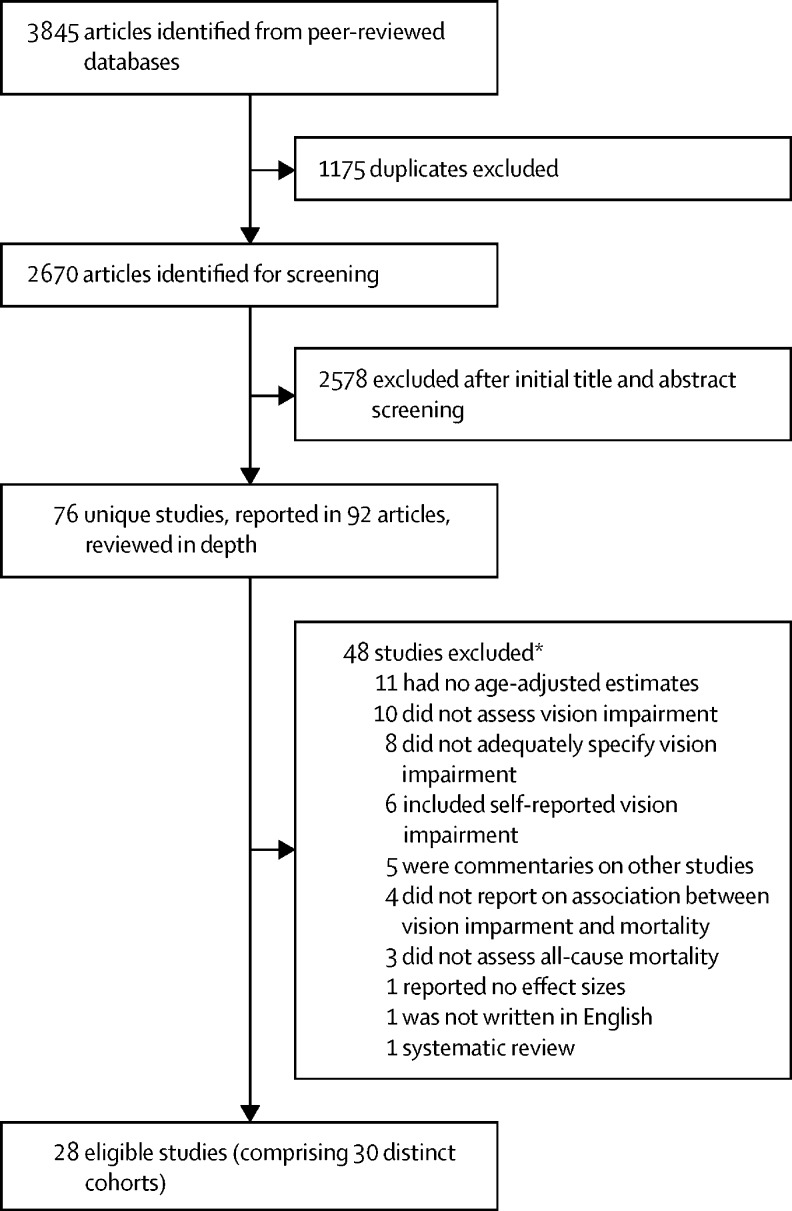
Study selection *Some studies were excluded for more than one reason.

We did a random-effects meta-analysis to generate a pooled effect estimate, reported as the hazard ratio (HR) with 95% CIs, for the association between vision impairment and age-adjusted, all-cause mortality. Between-study heterogeneity was assessed with *I*^2^ and τ^2^ statistics. The primary analysis used maximally adjusted estimates, and the sensitivity analysis used minimally adjusted estimates. For studies in which only one estimate was available, the same estimate was used in both the maximally adjusted and minimally adjusted models.

We did meta-regression analyses to test whether effect estimates varied by the following factors: risk of bias, type of visual acuity chart, analysis of better-eye data (compared with other definitions), the use of best-corrected visual acuity or presenting visual acuity as the vision assessment method, follow-up duration, a lower age limit (ie, participants aged <50 years *vs* those aged ≥50 years), and study design. We only did meta-regression analyses for studies that reported on the association between mortality and vision impairment, defined as a visual acuity of <6/12, as there were too few studies to do meta-regression analyses for other vision impairment categories. The results are not reported by Global Burden of Disease Study super-region^17^ as planned because all studies with a visual acuity <6/12 group were done in high-income countries. We assessed publication bias using Egger's test (threshold for significance p<0·05) and by inspection of funnel plots. All analyses were done with Stata software, version 16.0.

Two investigators (JREh and JREv) graded the overall certainty of the evidence using the Grading of Recommendations, Assessment, Development and Evaluations (GRADE) framework, modified for prognostic studies.^18^ This instrument is used to rate the certainty of evidence by considering risk of bias, imprecision, inconsistency, indirectness, and publication bias.

### Role of the funding source

The funders of the study had no role in study design, data collection, data analysis, data interpretation, or writing of the report.

## Results

We identified 3845 articles through electronic database searches. After removing duplicate references, we screened the titles and abstracts of 2670 articles. Of these, we identified 76 unique studies in 92 articles for full-text review, during which 48 studies were excluded, leaving 28 studies^5^, ^6^, ^7^, ^8^, ^9^, ^10^, ^19^, ^20^, ^21^, ^22^, ^23^, ^24^, ^25^, ^26^, ^27^, ^28^, ^29^, ^30^, ^31^, ^32^, ^33^, ^34^, ^35^, ^36^, ^37^, ^38^, ^39^, ^40^ that met the inclusion criteria. Two of the studies each included two distinct cohorts; therefore, these 28 studies comprised 30 distinct cohorts (446 088 participants) from 12 countries. 25 were observational cohort studies,^5^, ^6^, ^8^, ^9^, ^10^, ^21^, ^22^, ^23^, ^24^, ^25^, ^26^, ^27^, ^28^, ^29^, ^30^, ^31^, ^32^, ^33^, ^34^, ^35^, ^36^, ^37^, ^38^, ^39^, ^40^ and three were RCTs^7^, ^19^, ^20^ reporting an association between vision impairment and mortality independent of the trial interventions.

**Figure 2:**
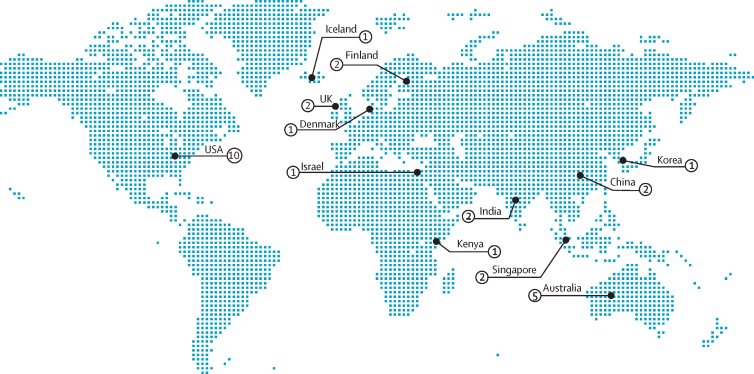
Global distribution of included studies The map shows the number of unique study cohorts from each country that were included in the systematic review.

The characteristics of each cohort are reported in table 1. The global distribution of the included cohorts is shown in figure 2; there were no cohorts from eastern Europe, Latin America, the Caribbean, north Africa, or the Pacific islands.

**Table 1:** Summary of included cohorts

	**Studies using the same cohort**	**Country**	**Sample size**	**Age, years**	**Ethnic group**	**Study design**	**Baseline assessment period**	**Mean follow-up, months**	**Visual acuity assessment instrument**	**Vision assessment method (eye)**	**Vision impairment definition**	**Mortality assessment method**
Agrawal et al (2011)^34^	NA	India	1422 (687 [48%] men and 735 [52%] women)	≥60	NR	Cohort	2008–09	17	Snellen chart	PVA (better eye)	<6/60	Death registry maintained by local health workers
Anstey et al (2001)^31^	NA	Australia	1947 (1039 [53%] men and 908 [47%] women)	≥70	NR	Cohort	1992–93	72	Snellen chart	BCVA (eye NR)	Various categories	Follow-up with participants and death certificates
Buch et al (2005)^38^	NA	Denmark	946 (462 [49%] men and 484 [51%] women)	60–80	NR	Cohort	1986–88	168	Snellen Tumbling E chart	BCVA (better eye)	≤6/12	National death registry
Clemons et al (2004)^20^	NA	USA	4753 (2099 [44%] men and 2654 [56%] women)	55–81	4546 (95%) white and 207 (5%) other	RCT	1992–98	78	ETDRS chart	BCVA (either eye)	<6/12	Death certificates and hospital records
Crewe et al (2015)^23^	NA	Australia	3452 (1350 [39%] men and 2102 [61%] women)	≥65	3231 (94%) non-Indigenous Australians, 15 (<1%) Indigenous Australians, and 208 (6%) unknown	Cohort	2003–10	123	NR	BCVA (better eye)	<6/60	National death registry
Fisher et al (2014)^39^	NA	Iceland	4926 (2121 [43%] men and 2805 [57%] women)	66–96	NR	Cohort	2002–06	64	Nidek ARK 760	PVA (better eye)	<6/12	National death registry
Foong et al (2008)^29^	NA	Singapore	1225 (553 [45%] men and 672 [55%] women)	40–79	NR	Cohort	1997–98	80	logMAR	BCVA (better eye)	<6/12	National death registry
Freeman et al (2005)^37^	Christ et al (2014)^41^	USA	1991 (836 [42%] men and 1155 [58%] women)	65–84	497 (26%) African American and 1494 (74%) white	Cohort	1993–2003	118	ETDRS chart	PVA (eye NR)	Per unit change	Follow-up with participants
Jacobs et al (2005)^33^	Stessman et al (2005)^42^	Israel	452 (245 [54%] men and 207 [46%] women)	70	NR	Cohort	1990	96	Snellen equivalent	BCVA (better eye)	≤6/12	National death registry
Karpa et al (2009)^30^	Gopinath et al (2013),^43^ Wang et al (2001),^44^ and Cugati et al (2007)^45^	Australia	3654 (1569 [43%] men, 2054 [57%] women, and 31 [1%] NR)	49–97	NR	Cohort	1991 and 1993	156	logMAR	PVA (better eye)	<6/12	National death registry
Khanna et al (2013)^27^	NA	India	4188 (1964 [47%] men and 2224 [53%] women)	30–70	NR	Cohort	1996–2000	132	logMAR	PVA (eye NR)	<6/18, <6/60	Key informants
Kim et al (2019)^6^	NA	South Korea	359 984 (195 052 [54%] men and 164 932 [46%] women)	≥40	NR	Cohort	2009–10	48	NR	BCVA (various)	<6/30, <6/300	National death registry
Knudtson et al (2006)^36^	Schubert et al (2017),^46^ Klein et al (2006),^47^ and Klein et al (1995)^48^	USA	4897 (42% men and 58% women)*	43–84	99% white and 1% NR*	Cohort	1987–88	158	ETDRS chart	BCVA (better eye)	≤6/12	National death registry and follow-up with participants
Kulmala et al (2008)^21^	Era et al (1997)^49^	Finland	223 (80 [36%] men and 143 [64%] women)	75	Finnish	Cohort	1989–90	106	Landolt C ring	PVA (better eye)	Various categories	National death registry and follow-up with participants
Kulmala et al (2008)^21^	Era et al (1997)^49^	Finland	193 (56 [29%] men and 137 [71%] women)	80	Finnish	Cohort	1989–90	89	Landolt C ring	PVA (better eye)	Various categories	National death registry and follow-up with participants
Kuper et al (2019)^5^	NA	Kenya	3441 (1656 [48%] men and 1785 [52%] women)	≥50	Kikuyu and Kalenjin	Cohort	2007–14	72	logMAR	PVA (better eye)	Various categories	Key informants
Lee et al (2003)^40^	NA	USA	245 (109 [44%] men and 136 [56%] women)	25–74	African American	Cohort	1974–75	210	Sloan chart	PVA (binocular)	<6/12	National death registry, death certificates, hospital records, insurance records, key informants, and follow-up with participants
Lee et al (2003)^40^	NA	USA	2571 (1115 [43%] men and 1456 [57%] women)	25–74	Non-Hispanic white	Cohort	1974–75	210	Sloan chart	PVA (binocular)	<6/12	National death registry, death certificates, hospital records, insurance records, key informants, and follow-up with participants
Li et al (2011)^26^	NA	China	5057 (2383 [47%] men and 2674 [53%] women)	50–96	NR	Cohort	2006–10	48	logMAR	BCVA (better eye)	<6/18, <6/60	Follow-up with participants and death certificates
Liao et al (2019)^8^	Zheng et al (2014)^50^	USA	2550 (1257 [49%] men and 1293 [51%] women)	≥60	1543 (61%) non-Hispanic white, 370 (15%) non-Hispanic Black, 487 (19%) Mexican American, and 156 (6%) other	Cohort	1999–2011	119†	Nidek ARK 760	PVA (better eye)	<6/12	National death registry
Loprinzi et al (2016)^9^	Zheng et al (2014)^50^	USA	1658 (800 [48%] men and 858 [52%] women)	40–85	76 (5%) Mexican American, 40 (2%) other Hispanic, 1313 (79%) non-Hispanic white, 154 (9%) non-Hispanic Black, and 75 (5%) other	Cohort	2003–04	92	Nidek ARK 760	BCVA (better eye)	<6/12	National death registry
Lott et al (2010)^25^	NA	USA	900 (414 [46%] men and 486 [54%] women)	58–102	NR	Cohort	1992–95	120	Bailey-Lovie chart	PVA (binocular)	<6/12, <6/20	National death registry, local obituaries, and key informants
Ng et al (2018)^10^	Liu et al (2017)^51^ and Estevez et al (2018)^52^	Australia	1257 (476 [38%] men and 781 [62%] women)	≥40	Indigenous Australian	Cohort	2005–08	120	Snellen Tumbling E chart	PVA (better eye)	<6/12	National death registry
Papudesu et al (2018)^7^	NA	USA	4203 (1816 [43%] men and 2387 [57%] women)	50–80	3997 (95%) non-Hispanic white and 206 (5%) other	RCT	2006–08	60†	ETDRS chart	BCVA (left eye)	<6/12	NR
Pedula et al (2006)^22^	Swindell et al (2010)^53^	USA	4932 (all women)	≥65	White	Cohort	1986–88	144	Bailey-Lovie chart	PVA (binocular)	≤6/12	Follow-up with participants and death certificates
Siantar et al (2015)^28^	Foong et al (2007)^54^	Singapore	3273 (1573 [48%] men and 170 [52%] women)	40–79	Malay	Cohort	2004–12	87†	logMAR	BCVA (better eye)	<6/12	National death registry
Taylor et al (2000)^35^	McCarty et al (2001)^55^	Australia	3271 (1511 [46%] men and 1760 [54%] women)	40–98	NR	Cohort	1992–94	60	ETDRS chart	BCVA (better eye)	≤6/12	National death registry
Thiagarajan et al (2005)^19^	NA	UK	13 569 (5279 [39%] men and 8290 [61%] women)	≥75	NR	RCT	1995–99	73†	Glasgow cards	PVA (binocular)	Various categories	National death registry
Thompson et al (1989)^24^	NA	UK	469‡	≥75	NR	Cohort	1981–87	60	Snellen chart	BCVA (better eye)	Various categories	Local vital statistics registry
Wang et al (2014)^32^	Xu et al (2009)^56^	China	4439 (1928 [44%] men and 2511 [56%] women)	40–101	Han Chinese	Cohort	2001–11	120	Snellen chart	BCVA (worse eye)	Per unit change	Follow-up with participants, key informants, physicians, and hospital records

**AR**K=auto refractometer or keratometer. BCVA=best-corrected visual acuity. ETDRS=Early Treatment Diabetic Retinopathy Study. logMAR=logarithm of the minimum angle of resolution. NA=not applicable. NR=not reported. PVA=presenting visual acuity. RCT=randomised controlled trial.

* The exact number of male, female, white, and non-white participants included in vision impairment analyses in this study was NR.

† Median follow-up.

‡ Number of men and women NR.

Studies collected data between the 1970s and 2012. The findings from six cohorts had been published since 2015.^5^, ^6^, ^7^, ^8^, ^9^, ^10^ The duration of follow-up among included studies ranged from 17 months to 210 months, with a mean of 103·3 (SD 46·4) months. Sample sizes ranged from 193 participants in Finland^21^ to 359 984 participants in Korea.^6^ 24 cohorts contained an approximately equal number of male and female participants. However, the study by Pedula and colleagues^22^ included female participants only, and five other cohorts comprised less than 40% male participants.^10^, ^19^, ^21^, ^23^ None of the included studies had less than 40% female participants, although one study did not report on the gender distribution of participants.^24^

20 publications reported the HR as an effect estimate. Eight publications reported odds ratios or risk ratios of death for a given follow-up period. Given the high mortality rates in most cohorts, these estimates of effect size could not be considered as equivalent. Thus, meta-analyses were done only with studies reporting HRs or incident rate ratios.

Measures of vision other than visual acuity were not commonly used. Several studies measured visual fields,^6^, ^23^, ^25^ contrast sensitivity,^22^, ^25^ colour vision,^25^ and stereopsis.^25^ In this subset of studies, contrast sensitivity impairment, peripheral field loss, and stereoacuity impairment were all significantly associated with an increased hazard for mortality in adjusted models. However, because of the small number of studies that assessed vision using these tests, only visual acuity was considered in this report.

Studies used a wide variety of instruments to assess visual acuity. The most commonly used vision charts were logarithm of the minimum angle of resolution (n=6),^5^, ^26^, ^27^, ^28^, ^29^, ^30^ Snellen charts (n=5),^24^, ^31^, ^32^, ^33^, ^34^ and Early Treatment Diabetic Retinopathy Study charts (n=5),^7^, ^20^, ^35^, ^36^, ^37^ whereas two studies did not specify the instrument used.^6^, ^23^ There was also considerable heterogeneity in the methods used to define vision impairment. 15 (54%) studies used best-corrected visual acuity to define vision impairment, and 17 (61%) studies defined vision impairment based on visual acuity in the better-seeing eye.

Definitions of vision impairment also varied between studies. Two studies reported the association between mortality and a continuous measure of visual acuity.^32^, ^37^ Six other studies (comprising seven cohorts) compared a reference group of participants with good vision with groups of participants with various non-overlapping vision impairment categories.^5^, ^19^, ^21^, ^24^, ^26^, ^31^ The remaining studies compared participants with visual acuity better than and worse than one or more visual acuity thresholds.

Studies used various strategies to assess mortality, and were included regardless of the methods used because official death registries might not have been available or provided high-quality data in many low-income and middle-income countries (LMICs).^57^ Most studies (n=24) searched official vital records, with some (n=12) also relying on other methods, including following up with participants, key informants, or both.

**Figure 3:**
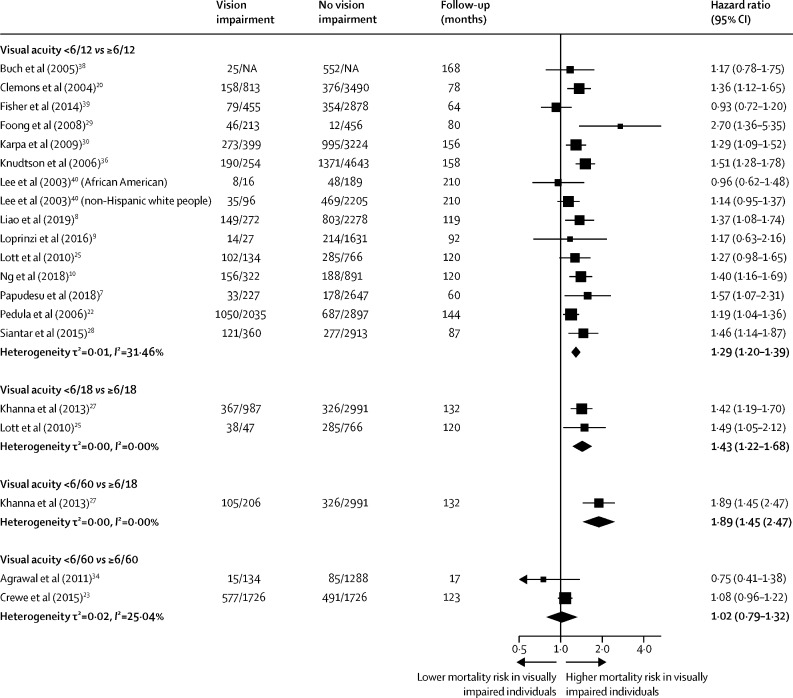
Random-effects meta-analysis results For each study, the number of participants who died out of the total number of participants in the study is shown. Data are the maximally adjusted pooled hazard ratios of mortality in adults with mild vision impairment or worse (visual acuity <6/12) versus those with a visual acuity of ≥6/12; moderate vision impairment or worse (visual acuity <6/18) versus those with a visual acuity of ≥6/18; and severe vision impairment or blindness (visual acuity <6/60) versus those with a visual acuity of ≥6/18 and ≥6/60. NA=not applicable.

The pooled maximally adjusted HRs for mortality in adults with vision impairment compared with those who had better vision are shown in figure 3. The 18 cohorts included in the meta-analysis comprised 47 998 participants. The remaining 12 cohorts identified in the systematic review were not included in the meta-analysis for one or more of the following reasons: they used a vision impairment threshold that could not be aggregated with other studies;^6^ they reported results per unit difference in visual acuity;^32^, ^37^ they reported measures of effect that could not be pooled with HRs;^5^, ^19^, ^24^, ^26^, ^31^, ^32^, ^33^, ^35^ or they compared a reference category of participants with good vision to participants with various non-overlapping vision impairment categories.^5^, ^19^, ^21^, ^24^, ^26^, ^31^ The associations between vision impairment and mortality among these 12 cohorts are shown in table 2.

**Table 2:** Results of studies not included in the meta-analysis

	**Reason for exclusion from the meta-analysis**	**Vision impairment definition (vision assessment method, eye)**	**Comparison group**	Minimally adjusted effect estimates (95% CI)*	**Maximally adjusted effect estimates (95% CI)**
Anstey et al (2001)^31^	Categorical analysis	6/9 (BCVA, eye NR); 6/12 (BCVA, eye NR); and 6/18 to 6/60 (BCVA, eye NR)	6/6	6/9, RR 0·95 (0·67–1·33); 6/12, 1·16 (0·84–1·59); and 6/18 to 6/60, 1·10 (0·80–1·53)	6/9, RR 0·89 (0·63–1·25); 6/12, 1·10 (0·80–1·52); and 6/18 to 6/60, 1·01 (0·72–1·39)
Freeman et al (2005)^37^	Effect estimate per unit change in visual acuity	Mild vision loss, 2–3 lines (PVA, eye NR); moderate vision loss, ≥3 lines (PVA, eye NR); and vision gain, ≥2 lines (PVA, eye NR)	No change in visual acuity	Mild vision loss, HR 0·92 (0·61–1·37); moderate vision loss, 2·23 (1·43–3·46); and vision gain, HR 0·47 (0·23–0·96)	Mild vision loss, HR 0·91 (0·61–1·36); moderate vision loss, 2·26 (1·45–3·52); and vision gain, 0·47 (0·23–0·95)
Jacobs et al (2005)^33^	Did not report HRs	≤6/12 (BCVA, better eye)		NA	OR 2·84 (1·48–5·46)
Kim et al (2019)^6^	Non-standard vision impairment thresholds	Mild vision loss, 6/30 to 6/100 (BCVA, better eye) or ≤6/300 (BCVA, worse eye); and severe vision loss, ≤6/300 (BCVA, better eye)	>6/30	Mild vision loss, HR 1·17 (0·81–1·69); and severe vision loss, 1·90 (1·08–3·35)	Mild vision loss, HR 1·16 (0·81–1·67); and severe vision loss, 1·87 (1·06–3·29)
Kulmala et al (2008)^21^; 75-year-old cohort	Categorical analysis	≤6/12 to ≥6/18 (PVA, better eye); and <6/18 (PVA, better eye)	>6/12	≤6/12 to ≥6/18, HR 1·98 (1·25–3·13); and <6/18, 1·90 (1·12–3·20)	≤6/12 to ≥6/18, HR 2·11 (1·27–3·48); and <6/18, 1·34 (0·75–2·39)
Kulmala et al (2008)^21^; 80-year-old cohort	Categorical analysis	≤6/12 to ≥6/18 (PVA, better eye); and <6/18 (PVA, better eye)	>6/12	≤6/12 to ≥6/18, HR 1·13 (0·74–1·72); and <6/18, 0·92 (0·47–1·78)	≤6/12 to ≥6/18, HR 0·77 (0·48–1·26); and <6/18, 0·75 (0·33–1·67)
Kuper et al (2019)^5^	Categorical analysis and did not report HRs	<6/12 to ≥6/18 (PVA, better eye); <6/18 to ≥6/60 (PVA, better eye); and <6/60 (PVA, better eye)	≥6/12	<6/12 to ≥6/18, RR 0·92 (0·57–1·50); <6/18 to ≥6/60, 1·75 (1·28–2·40); and <6/60, 1·98 (1·04–3·80)	<6/12 to ≥6/18, RR 0·82 (0·48–1·41); <6/18 to ≥6/60, 1·56 (1·14–2·15); and <6/60, 1·46 (0·80–2·68)
Li et al (2011)^26^	Categorical analysis and did not report HRs	<6/18 to ≥3/60 (BCVA, better eye); <3/60 (BCVA, better eye)	≥6/18	NA	<6/18 to ≥3/60, OR 3·1 (1·5–6·4); and <3/60, 3·9 (2·1–7·2)
Taylor et al (2000)^35^	Did not report HRs	≤6/12 (BCVA, better eye)	>6/12	NA	OR 2·42 (1·07–5·43)
Thiagarajan et al (2005)^19^	Categorical analysis and did not report HRs	<6/6 to ≥6/9 (PVA, binocular); <6/9 to ≥6/18 (PVA, binocular); and <6/18 (PVA, binocular)	≥6/6	<6/6 to ≥6/9, RR 1·10 (1·01–1·19); <6/9 to ≥6/18, 1·32 (1·22–1·42); and <6/18, 1·60 (1·47–1·74)	<6/6 to ≥6/9, RR 1·06 (0·97–1·16); <6/9 to ≥6/18, 1·24 (1·14–1·35); and <6/18, 1·52 (1·39–1·66)
Thompson et al (1989)^24^	Categorical analysis and did not report HRs	≤6/7·5 to ≥6/9 (BCVA, better eye); ≤6/12 to ≥6/18 (BCVA, better eye); ≤6/24 to ≥6/60 (BCVA, better eye); and <6/60 (BCVA, better eye)	≥6/6	NA	≤6/7·5 to ≥6/9, RR 1·62 (0·87–3·01); ≤6/12 to ≥6/18, 1·83 (0·93–3·63); ≤6/24 to ≥6/60, 1·72 (0·77–3·84); and <6/60, 0·35 (0·08–1·57)
Wang et al (2014)^32^	Effect estimate per unit difference in visual acuity and did not report HRs	(BCVA, worse eye)	NA	OR 1·76 (1·35–2·29)	NA

The table shows effect estimates of studies that were excluded from meta-analysis, with reasons for exclusion and definitions of vision impairment. BCVA=best-corrected visual acuity. HR=hazard ratio. NA=not applicable. NR=not reported. OR=odds ratio. PVA=presenting visual acuity. RR=risk ratio.

* All estimates are, at minimum, adjusted for age.

A total of 14 studies (comprising 15 cohorts) compared the hazard for mortality in participants with a visual acuity of <6/12 versus those with a visual acuity of ≥6/12; the adjusted HR estimate for mortality was 1·29 (95% CI 1·20–1·39) and heterogeneity between studies was low (τ^2^=0·01, *I*^2^=31·46%), suggesting a consistent effect across studies (figure 3). Two studies compared the hazard for mortality among participants with a visual acuity <6/18 versus those with a visual acuity of ≥6/18; the adjusted estimated HR for mortality was 1·43 (1·22–1·68) and heterogeneity between studies was low (τ^2^=0·00, *I*^2^=0·00%). Only one study compared the hazard for mortality in participants with a visual acuity of <6/60 versus those with a visual acuity of ≥6/18, with a HR for mortality of 1·89 (1·45–2·47). Two studies compared the hazard for mortality in participants with a visual acuity of <6/60 versus those with a visual acuity of ≥6/60; the adjusted pooled HR for mortality was 1·02 (0·79–1·32; τ^2^=0·02, *I*^2^=25·04%).

The pooled minimally adjusted HR for mortality among participants with a visual acuity of <6/12 versus those with a visual acuity of ≥6/12 is shown in the appendix (p 8). In this analysis, the pooled minimally adjusted HR for mortality was 1·41 (95% CI 1·29–1·53).

**Figure 4:**
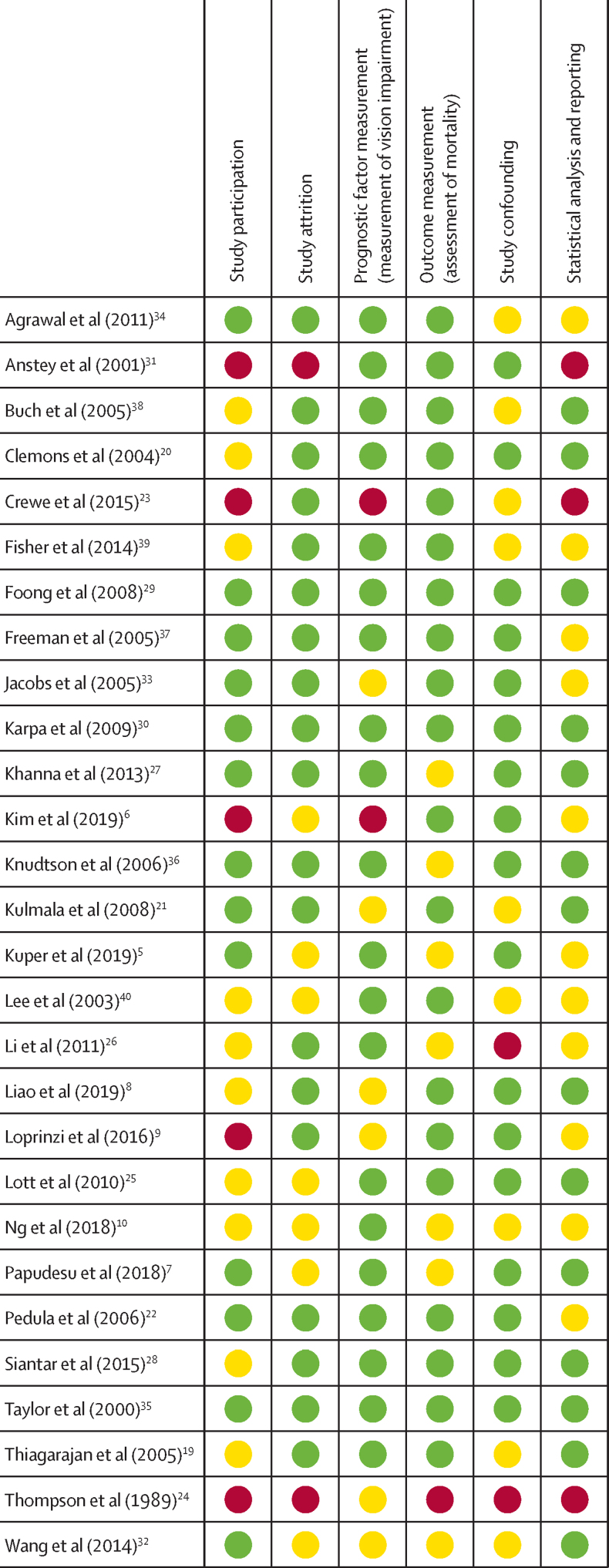
Risk of bias assessment Risk of bias was assessed by use of the Quality in Prognostic Studies tool.^16^ Green represents low risk of bias, yellow represents a moderate risk of bias, and red represents high risk of bias.

For risk of bias assessment using the QUIPS tool,^16^ only three studies received an assessment of low risk of bias across all six domains (figure 4).^29^, ^30^, ^35^ Six studies were assessed as having a high risk of bias in one or more domains.^6^, ^9^, ^23^, ^24^, ^26^, ^31^

Funnel plots were reviewed for studies comparing all-cause mortality in participants with a visual acuity of <6/12 with those that had a visual acuity of ≥6/12, and no evidence of publication bias was identified (p=0·63; appendix p 9). Meta-regression analysis of studies comparing all-cause mortality between these two groups of participants revealed no evidence that the estimated effect size differed by risk of bias, the type of vision chart used, the eye assessed, follow-up duration, a lower age limit (ie, participants aged <50 years *vs* those aged ≥50 years), or study design (table 3). However, studies that used best-corrected visual acuity as the vision assessment method reported a significantly higher hazard for mortality (HR 1·45 [95% CI 1·31–1·60]) than those using presenting visual acuity (1·22 [1·13–1·31]; p=0·0055).

**Table 3:** Meta-regression analysis results

		**Number of studies**	**HR (95% CI)**	**p value**
Risk of bias		..	..	0·76
	Low	2	1·73 (0·85–3·50)	..
	Moderate	12	1·28 (1·18–1·39)	..
	High	1	1·17 (0·63–2·16)	..
Vision chart		..	..	0·082
	ETDRS chart	3	1·46 (1·29–1·64)	..
	Snellen Tumbling E chart	2	1·35 (1·14–1·61)	..
	logMAR*	5	1·28 (1·17–1·40)	..
	Other or not reported	5	1·12 (0·95–1·31)	..
Eye		..	..	0·24
	Better eye	9	1·34 (1·19–1·50)	..
	Other	6	1·22 (1·12–1·33)	..
Vision assessment method		..	..	0·0055
	Best-corrected visual acuity	7	1·45 (1·31–1·60)	..
	Presenting visual acuity	8	1·22 (1·13–1·31)	..
Follow-up duration, years		..	..	0·58
	<10	7	1·35 (1·13–1·60)	..
	≥10	8	1·27 (1·17–1·39)	..
Lower age limit, years		..	..	0·27
	<50	7	1·35 (1·19–1·54)	..
	≥50	8	1·24 (1·15–1·34)	..
Study design		..	..	0·38
	Cohort	13	1·27 (1·17–1·39)	..
	Randomised controlled trial	2	1·40 (1·18–1·66)	..

Estimated HRs for mortality in people with a visual acuity of <6/12 versus those with a visual acuity of ≥6/12, subcategorised by seven variables, with p values from the meta-regression analysis. ETDRS=Early Treatment Diabetic Retinopathy Study. HR=hazard ratio. logMAR=logarithm of minimum angle of resolution.

* Includs Bailey-Lovie charts.

Using the GRADE framework, we judged the evidence to be of moderate certainty overall, downgrading half a level for risk of bias and half a level for inconsistency. Even though only three of the 28 studies were judged as having a low risk of bias in all domains, meta-regression analyses suggested that the effect estimates were not associated with risk of bias. Measured inconsistency or heterogeneity between studies was not high, but there was some variation in study results. Using this framework, we are moderately confident that the mortality risk associated with vision impairment reported in this study is likely to be close to the true value.^18^

## Discussion

This systematic review and meta-analysis summarises the existing evidence on the association between vision impairment and the risk of mortality among adults from 12 countries across five continents. The results support the existence of a consistent association between poor vision and mortality across different study settings, thereby reinforcing the specific importance of vision and eye health to Sustainable Development Goal (SDG) 3, which aims to “ensure healthy lives and promote well-being for all at all ages”, as well as to the SDGs more generally.^58^

This study builds on a previous meta-analysis that considered studies on the association between vision impairment and mortality published before 2015.^4^ This previous meta-analysis provided evidence that vision impairment could be associated with an increased risk of mortality; however, the study also had several key limitations that we sought to address in the current report. First, the meta-analysis included studies that not only assessed vision with objective clinical measures (eg, visual acuity), but also self-reported visual difficulty, and administrative billing codes. Because of the heterogeneous data, the study compared participants with the highest level of vision impairment to those with no vision impairment. This approach could have resulted in misclassification bias, since poor visual acuity, self-reported visual difficulty, and ICD codes could represent distinct constructs. In addition, this approach could have overestimated effect sizes by only including participants in the best and worst vision categories. However, in the meta-regression analyses, the effect size was similar for the 15 studies that assessed vision with visual acuity charts (relative risk 1·36 [95% CI 1·16–1·59]), for the three studies that used ICD codes (1·55 [1·15–2·09]), and for the seven studies that used self-reported visual acuity (1·44 [1·34–1·56]). Another limitation of this previous meta-analysis was not assessing the risk of bias in included studies or not including an overall assessment of the certainty of the evidence.

The meta-analyses in our study help to quantify the magnitude of the association between vision impairment and mortality. The hazard for mortality was 29% higher for participants with a visual acuity of <6/12 versus those with a visual acuity of ≥6/12, 43% higher for participants with a visual acuity of <6/18 versus those with a visual acuity of ≥6/18, and 89% higher for participants with a visual acuity of <6/60 versus those with a visual acuity of ≥6/18. However, there was no significant difference in the hazard for mortality between participants with a visual acuity of <6/60 versus those with a visual acuity of ≥6/60, probably because the reference group (visual acuity ≥6/60) in these studies contained participants with a substantial degree of vision impairment. Data from 12 cohorts identified in the systematic review could not be included in the meta-analyses because they were not comparable with other included studies in terms of their analytical methods and categorisation of vision impairment. Among these heterogeneous cohorts, most (n=9) reported a significant association between vision impairment and increased mortality.

Notably, there was considerable variability in the methods used to assess and report visual acuity and mortality among included studies, which could affect interpretation of the findings. However, results of meta-regression analyses showed that the hazard for mortality was not significantly affected by the eye chart used to assess visual acuity or the eye used to assess the level of vision impairment (eg, the better-seeing eye). Nonetheless, the high degree of variability in the measurement and reporting of visual acuity data does highlight the need for widespread adoption of standard definitions and protocols to promote comparability across cohorts in future studies. *The Lancet Global Health* Commission on Global Eye Health has proposed visual acuity measurement and reporting standards for epidemiological studies, which are described in detail in the main Commission report.^11^

Meta-regression analysis revealed that the hazard for mortality was significantly higher in studies reporting best-corrected visual acuity compared with those reporting presenting visual acuity as the vision assessment method. This finding suggests that, compared with uncorrected refractive error, non-refractive causes of vision impairment might have a stronger association with mortality. This association could be due to common risk factors for non-refractive vision loss and mortality (eg, stroke or diabetes). Non-refractive vision impairment could also have a greater effect on factors that mediate the association with mortality (eg, physical activity). It is also possible that some study participants with vision impairment due to uncorrected refractive error received glasses during the course of the study, which could have decreased their risk of vision-impairment-related mortality. Additionally, some causes of vision impairment, such as cataract, glaucoma, and age-related macular degeneration, have been referred to as markers of ageing and might therefore indicate accelerated biological ageing.^36^

This systematic review and meta-analysis was limited by the wide variation in how studies adjusted for potential confounding variables, which could have biased the findings. Nonetheless, both maximally adjusted and minimally adjusted pooled effect estimates showed a significant association between vision impairment (visual acuity <6/12) and all-cause mortality. Since age is a strong common risk factor for both vision impairment and mortality, studies were only included if they reported, at a minimum, age-adjusted mortality. Studies also adjusted for other important factors that could confound the association between vision impairment and mortality. For example, socioeconomic deprivation, poor access to health care, diabetes, and stroke are a few of the well documented common risk factors for vision impairment and mortality,^3^ for which models were adjusted in many studies. Some studies, however, might have over-adjusted their statistical models, including for variables that might lie on the causal pathway between vision impairment and mortality. Adjusting for variables hypothesised to be on this causal pathway could bias study results toward the null hypothesis (ie, no effect).

Most included studies were from high-income countries, and additional evidence from regions not represented in the literature would contribute to a more complete understanding of this topic to inform policy. Future studies could also consider adopting standardised measurement and reporting guidelines, as outlined in the main Commission report.^11^ Furthermore, there is a need for studies to consider the risk of mortality associated with other types of vision impairment that are less commonly assessed, such as contrast sensitivity impairment and peripheral field loss. Mediating pathways between vision impairment and mortality, which could include shared risk factors, such as physical inactivity, social isolation, and disability,^3^, ^59^, ^60^, ^61^ should be investigated. Finally, future calculations of disability-adjusted life-years might include years of life lost due to vision impairment, which could provide a more complete estimate of the overall global burden of vision impairment.

The current study has several key strengths. First, we included multiple additional studies published in 2016–19,^5^, ^6^, ^7^, ^8^, ^9^, ^10^ including those from regions of the world that were not well represented in the previous meta-analysis,^4^ such as sub-Saharan Africa (Kenya)^5^ and east Asia (South Korea).^6^ Second, we only included studies that assessed visual acuity. Even though this strategy might have resulted in some well designed studies being excluded, it served to strengthen the internal validity of our meta-analysis and to limit misclassification bias. The current investigation also included an assessment of the risk of bias in included studies using the well described QUIPS tool.^16^ Even though only three included studies were considered to have a low risk of bias across all domains, there was no evidence from the meta-regression analyses that the estimated association was affected by risk of bias. Finally, by use of the GRADE framework, an overall assessment of the strength of the evidence was described.

The results of this study have important implications for policy and practice. Worldwide, more than 80% of people with vision impairment and blindness live in LMICs, and 55% are women and girls.^1^ Four of five cases of vision impairment and blindness are preventable or correctable. In fact, the leading causes of vision impairment and blindness worldwide are cataract and uncorrected refractive error,^62^ both of which are readily treatable with inexpensive, cost-effective interventions.^63^ Therefore, there is an important opportunity to promote not only health and wellbeing, but also longevity by correcting, rehabilitating, and preventing avoidable vision loss. Policies and strategies for achieving this are outlined in the main Commission report,^11^ and have the potential to make important contributions to achieving the SDGs.

## Data sharing

The protocol for this study has been published previously. As this study is a systematic review and meta-analysis, there are no primary data to be shared.
