# Supplementary material for: Association between vision impairment and mortality: a systematic review and meta-analysis
Source: Lancet Glob Health. 2021 Feb 16;9(4):e418–30. doi: 10.1016/S2214-109X(20)30549-0 (PMC7966688; doi:10.1016/S2214-109X(20)30549-0)
Supplement: Supplementary appendix [file mmc1.pdf]

THE LANCET
Global Health

Supplementary appendix

This appendix formed part of the original submission and has been peer reviewed. We post it as supplied by the authors.

Supplement to: Ehrlich JR, Ramke J, Macleod D, et al. Association between vision impairment and mortality: a systematic review and meta-analysis. *Lancet Glob Health* 2021; published online Feb 16. http://dx.doi.org/10.1016/S2214-109X(20)30549-0.

APPENDIX 1.

Contents

Full Search Terms… pages 2-5

APPENDIX 2.

Contents

Figure 1. PRISMA Checklist… pages 6-7

APPENDIX 3.

Contents

Figure 1. Random-effects meta-analysis results showing the minimally-adjusted pooled hazard of mortality… page 8

Figure 2. Funnel plot… page 9

APPENDIX 1. Full Search Terms

Text 1. MEDLINE

1. exp eye diseases/
2. Visually Impaired Persons/
3. ((low$ or handicap$ or subnormal$ or impair$ or partial$ or disab$ or disorder$ or loss$ or limit$) adj3 (vision or visual$ or sight$)).tw.
4. ((macul$ or retina$ or choroid$) adj3 degener$).tw.
5. ((macul$ or retina$ or choroid$) adj3 neovasc$).tw.
6. (AMD or ARMD).tw.
7. ((diabet$ or proliferative or non-proliferative or pre-proliferative) adj4 retinopath$).tw.
8. (diabet$ adj2 (eye$ or vision or visual$ or sight$)).tw.
9. glaucoma$.tw.
10. cataract$.tw.
11. blindness.tw.
12. Visual Acuity/
13. visual acuit$.tw.
14. Contrast Sensitivity/
15. (contrast adj2 sensitivity).tw.
16. Depth Perception/
17. stereopsis.tw.
18. (stereo adj1 acuit$).tw.
19. Visual Fields/
20. ((visual$ or vision) adj2 function$).tw.
21. or/1-20
22. exp Mortality/
23. Death Certificates/
24. mortality.tw.
25. death$.tw.
26. (fatality or fatalities).tw.
27. or/22-26
28. Cohort Studies/
29. Longitudinal Studies/
30. (cohort$ or longitudinal).tw.
31. Cross-Sectional Studies/
32. "Surveys and Questionnaires"/

33. Health Surveys/

34. (survey or surveys).tw.

35. or/28-34

36. 21 and 27 and 35

37. (neonate$ or preterm$ or prematurity or infant$ or child$).tw.

38. visual analog$ scale.tw.

39. (case adj2 report$).tw.

40. (animal or mouse or mice or rat or rats).ti.

41. or/37-40

42. 36 not 41

Text 2. Embase

1. exp eye disease/co, cn, di, dm, dr, dt, ep, et, pc, rt, rh, su, th [Complication, Congenital Disorder, Diagnosis, Disease Management, Drug Resistance, Drug Therapy, Epidemiology, Etiology, Prevention, Radiotherapy, Rehabilitation, Surgery, Therapy]

2. exp visual impairment/

3. ((low$ or handicap$ or subnormal$ or impair$ or partial$ or disab$ or disorder$ or loss$ or limit$) adj3 (vision or visual$ or sight$)).tw.

4. ((macul$ or retina$ or choroid$) adj3 degener$).tw.

5. ((macul$ or retina$ or choroid$) adj3 neovasc$).tw.

6. (AMD or ARMD).tw.

7. ((diabet$ or proliferative or non-proliferative or pre-proliferative) adj4 retinopath$).tw.

8. (diabet$ adj2 (eye$ or vision or visual$ or sight$)).tw.

9. glaucoma$.tw.

10. cataract$.tw.

11. blindness.tw.

12. visual acuity/

13. visual acuit$.tw.

14. contrast sensitivity/

15. (contrast adj2 sensitivity).tw.

16. stereoscopic vision/

17. stereopsis.tw.

18. (stereo adj1 acuit$).tw.

19. visual field defect/

20. visual field/

21. ((visual$ or vision) adj2 function$).tw.

22. or/1-21

23. exp mortality/

24. death/

25. death certificate/

26. mortality.tw.

27. death$.tw.

28. (fatality or fatalities).tw.

29. or/23-28

30. cohort analysis/

31. longitudinal study/

32. (cohort$ or longitudinal).tw.

33. cross-sectional study/

34. questionnaire/

35. health survey/ or health care survey/

36. (survey or surveys).tw.

37. or/30-36

38. 22 and 29 and 37

39. (neonate$ or preterm$ or prematurity or infant$ or child$).tw.

40. visual analog$ scale.tw.

41. (case adj2 report$).tw.

42. (animal or mouse or mice or rat or rats).ti.

43. or/39-42

44. 38 not 43

45. limit 44 to conference abstract status

46. 44 not 45

Text 3. Global Health

1. eye diseases/

2. vision/

3. vision disorders/

4. ((low$ or handicap$ or subnormal$ or impair$ or partial$ or disab$ or disorder$ or loss$ or limit$) adj3 (vision or visual$ or sight$)).tw.

5. ((macul$ or retina$ or choroid$) adj3 degener$).tw.

6. ((macul$ or retina$ or choroid$) adj3 neovasc$).tw.

7. (AMD or ARMD).tw.

8. ((diabet$ or proliferative or non-proliferative or pre-proliferative) adj4 retinopath$).tw.

9. (diabet$ adj2 (eye$ or vision or visual$ or sight$)).tw.

10. glaucoma$.tw.

11. cataract$.tw.

12. blindness.tw.

13. visual acuit$.tw.

14. (contrast adj2 sensitivity).tw.

15. stereopsis.tw.

16. (stereo adj1 acuit$).tw.

17. ((visual$ or vision) adj2 function$).tw.

18. or/1-17

19. mortality/

20. death/

21. "causes of death"/

22. mortality.tw.

23. death$.tw.

24. (fatality or fatalities).tw.

25. or/19-24

26. cohort studies/

27. longitudinal studies/

28. (cohort$ or longitudinal).tw.

29. questionnaires/

30. surveys/

31. (survey or surveys).tw.

32. or/26-31

33. 18 and 25 and 32

34. (neonate$ or preterm$ or prematurity or infant$ or child$).tw.

35. visual analog$ scale.tw.

36. (case adj2 report$).tw.

37. (animal or mouse or mice or rat or rats).ti.

38. or/34-37

39. 33 not 38

APPENDIX 2. PRISMA Checklist

Figure 1.

PRISMA 2009 Checklist

Section/topic	#	Checklist item	Reported on page #
**TITLE**			
Title	1	Identify the report as a systematic review, meta-analysis, or both.	1
**ABSTRACT**			
Structured summary	2	Provide a structured summary including, as applicable: background; objectives; data sources; study eligibility criteria, participants, and interventions; study appraisal and synthesis methods; results; limitations; conclusions and implications of key findings; systematic review registration number.	2
**INTRODUCTION**			
Rationale	3	Describe the rationale for the review in the context of what is already known.	3
Objectives	4	Provide an explicit statement of questions being addressed with reference to participants, interventions, comparisons, outcomes, and study design (PICOS).	4
**METHODS**			
Protocol and registration	5	Indicate if a review protocol exists, if and where it can be accessed (e.g., Web address), and, if available, provide registration information including registration number.	4
Eligibility criteria	6	Specify study characteristics (e.g., PICOS, length of follow-up) and report characteristics (e.g., years considered, language, publication status) used as criteria for eligibility, giving rationale.	4
Information sources	7	Describe all information sources (e.g., databases with dates of coverage, contact with study authors to identify additional studies) in the search and date last searched.	4
Search	8	Present full electronic search strategy for at least one database, including any limits used, such that it could be repeated.	5
Study selection	9	State the process for selecting studies (i.e., screening, eligibility, included in systematic review, and, if applicable, included in the meta-analysis).	4
Data collection process	10	Describe method of data extraction from reports (e.g., piloted forms, independently, in duplicate) and any processes for obtaining and confirming data from investigators.	5
Data items	11	List and define all variables for which data were sought (e.g., PICOS, funding sources) and any assumptions and simplifications made.	5
Risk of bias in individual studies	12	Describe methods used for assessing risk of bias of individual studies (including specification of whether this was done at the study or outcome level), and how this information is to be used in any data synthesis.	5
Summary measures	13	State the principal summary measures (e.g., risk ratio, difference in means).	5
Synthesis of results	14	Describe the methods of handling data and combining results of studies, if done, including measures of consistency (e.g., I^2) for each meta-analysis.	5
Risk of bias across studies	15	Specify any assessment of risk of bias that may affect the cumulative evidence (e.g., publication bias, selective reporting within studies).	5
Additional analyses	16	Describe methods of additional analyses (e.g., sensitivity or subgroup analyses, meta-regression), if done, indicating which were pre-specified.	5

Section/topic	#	Checklist item	Reported on page #
**RESULTS**			
Study selection	17	Give numbers of studies screened, assessed for eligibility, and included in the review, with reasons for exclusions at each stage, ideally with a flow diagram.	6
Study characteristics	18	For each study, present characteristics for which data were extracted (e.g., study size, PICOS, follow-up period) and provide the citations.	6
Risk of bias within studies	19	Present data on risk of bias of each study and, if available, any outcome level assessment (see item 12).	7
Results of individual studies	20	For all outcomes considered (benefits or harms), present, for each study: (a) simple summary data for each intervention group (b) effect estimates and confidence intervals, ideally with a forest plot.	7
Synthesis of results	21	Present results of each meta-analysis done, including confidence intervals and measures of consistency.	7
Risk of bias across studies	22	Present results of any assessment of risk of bias across studies (see Item 15).	7
Additional analysis	23	Give results of additional analyses, if done (e.g., sensitivity or subgroup analyses, meta-regression [see Item 16]).	7
**DISCUSSION**			
Summary of evidence	24	Summarize the main findings including the strength of evidence for each main outcome; consider their relevance to key groups (e.g., healthcare providers, users, and policy makers).	8
Limitations	25	Discuss limitations at study and outcome level (e.g., risk of bias), and at review-level (e.g., incomplete retrieval of identified research, reporting bias).	8
Conclusions	26	Provide a general interpretation of the results in the context of other evidence, and implications for future research.	9
**FUNDING**			
Funding	27	Describe sources of funding for the systematic review and other support (e.g., supply of data); role of funders for the systematic review.	2 & 6

From: Moher D, Liberati A, Tetzlaff J, Altman DG, The PRISMA Group (2009). Preferred Reporting Items for Systematic Reviews and Meta-Analyses: The PRISMA Statement. PLoS Med 6(7): e1000097. doi:10.1371/journal.pmed1000097

For more information, visit: **www.prisma-statement.org**.

APPENDIX 3.

Figure 1. Random-effects meta-analysis results showing the minimally-adjusted pooled hazard of mortality in adults with mild vision impairment or worse.

Study	VI N	VI Events	Not VI N	Not VI Events	Follow-up (Months)	Hazard ratio (95% CI)
Buch 2005	.	25	.	552	168	1.17 (0.78, 1.75)
Clemons 2004	813	158	3940	376	78	1.65 (1.36, 2.00)
Fisher 2014	455	79	2878	354	64	1.02 (0.79, 1.31)
Foong 2008	213	46	456	12	80	3.20 (1.72, 5.96)
Karpa 2009	399	273	3224	995	156	1.49 (1.29, 1.73)
Knudtson 2006	254	190	4643	1371	158	1.42 (1.19, 1.69)
Lee 2003 (African American)	16	8	189	48	210	0.89 (0.48, 1.66)
Lee 2003 (non-Hispanic whites)	96	35	2205	469	210	1.16 (1.02, 1.32)
Liao 2019	272	149	2278	803	119	1.43 (1.18, 1.73)
Loprinzi 2016	27	14	1631	214	92	1.17 (0.63, 2.16)
Lott 2010	134	102	766	285	120	1.27 (0.98, 1.65)
Ng 2018	322	156	891	188	120	1.40 (1.16, 1.69)
Papudesu 2018	227	33	2647	178	60	1.57 (1.08, 2.29)
Pedula 2006	2035	1050	2897	687	144	1.58 (1.42, 1.76)
Siantar 2015	360	121	2913	277	87	1.67 (1.32, 2.11)
**Overall**						1.41 (1.29, 1.53)

Heterogeneity: $\tau^2 = 0.01$, $I^2 = 55.01\%$

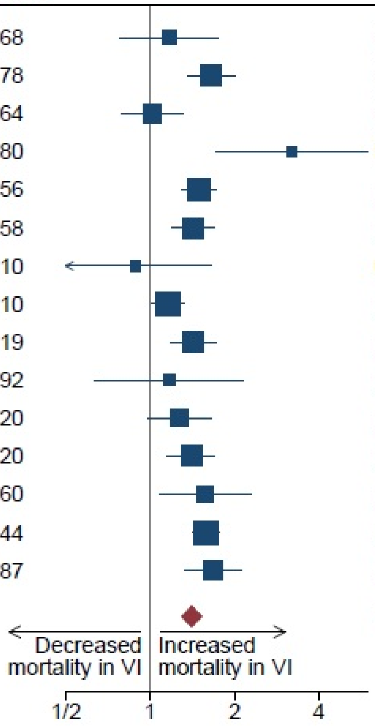

Events are defined as the number of participants in the study who died and n is the total number of participants in the study.

CI: confidence interval, VI: vision impairment

Figure 2. Funnel plot for studies assessing all-cause mortality in adults with mild vision impairment or worse (<6/12 compared with ≥6/12)

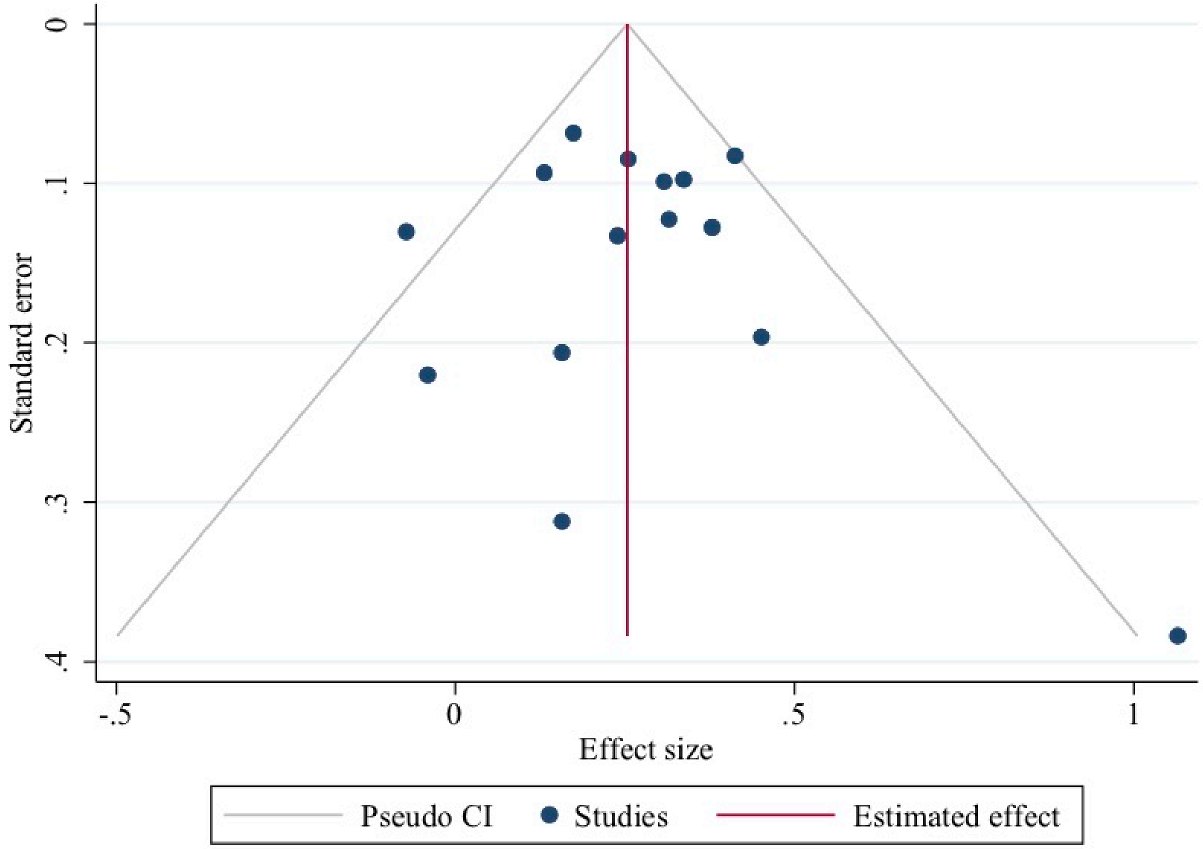

CI: confidence interval
